# Tumour to Tumour Metastasis of Malignant Melanoma to Intracranial Tumour

**DOI:** 10.4021/jocmr2009.11.1273

**Published:** 2009-12-28

**Authors:** Zakir Shariff, Phil Lim, Andrew Wright, Sharif Al-Ghazal

**Affiliations:** a Department of Plastic Surgery, Bradford Royal Infirmary, Bradford, UK

## Abstract

Tumours can metastasize to sites of other tumours but this is a rare event. The phenomenon has been well described in primary tumours of the lung and breast. This has been described in melanomas but as very rare occurrence. We describe a case of a malignant melanoma that metastasized to a pre-existing meningioma. We have tried to highlight this phenomenon in patient with melanoma and need for close vigilance of other pre-existing tumours to identify early metastasis.

**Keywords:**

Melanoma; Metastasis; Tumour to Tumor; Intracranial; Meningioma

## Introduction

Tumor-to-tumor metastasis is a rare phenomenon. The most common donors of tumor-to-tumor metastasis are carcinomas of the lung, followed by carcinomas of the breast, gastrointestinal tract, prostate, and thyroid [1]. Melanoma is the third most common cancer to metastasis to brain [2] but metastasis into meningoma is very rare.

Meningiomas have a very vascular architecture that may be susceptible to deposition of metastasis from extracranial tumours though anaplastic primary brain tumours are more vascular and metastases there is rarer. The phenomenon has been described most commonly with primary tumours of the lung and breast though more rarely metastasis from kidney and thyroid can occur.

## Case Report

A 51 years old Caucasian patient presented with a pigmented lesion on her right arm which had changed recently. Histology of the excised lesion revealed a superficial spreading melanoma of BT 2.9mm and Clarkes' Level IV. Patient developed swelling in the right axilla, 9 months later. Axillary dissection performed, showed metastatic melanoma in 7/18 nodes without extra-capsular spread. She had previously been diagnosed with a sphenoid wing meningioma 3 years prior to the diagnosis of the melanoma. This had been managed conservatively with regular repeat CT scans.

Six months following axillary dissection, she suffered an acute neurological event resulting in right sided weakness and difficulty in speaking resulting.

**Figure 1:**
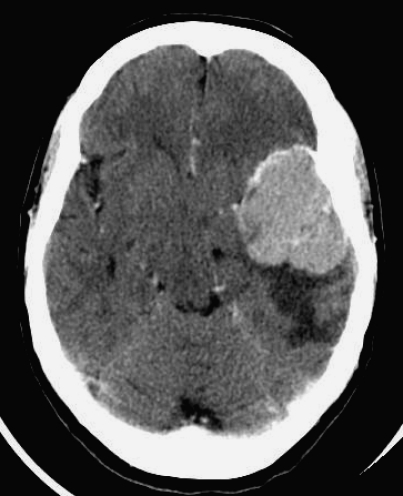
Meningioma arising from Sphenoid wing with melanoma metastasis within.

**Figure 2:**
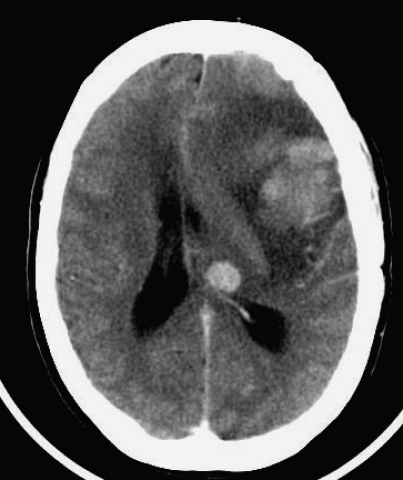
Post operative CT with intracranial haemorrhage and recurrence of the tumour.

A CT scan was performed and this revealed the presence of meningoma that had increased in size (Fig. 1). A subsequent scan after further deterioration demonstrated a midline shift with haemorrhage into the left temporal lobe (Fig. 2). She was transferred to the regional neurosurgical unit where the meningioma was resected. Histology showed the presence of metastatic melanoma within the meningioma. The patient underwent a programme of rehabilitation but unfortunately rapidly deteriorated a month later. A further CT scan then showed inoperable intracranial metastasis and she had palliation of her symptoms before dying 2 months later.

## Discussion

Metastasis of one tumour to another tumour is a rare occurrence. Campbell et aL proposed strict criteria for “true” tumour-to-tumour metastasis based on previously published work, and the following four criteria should be met: (i) at least two primary tumours exist; (ii) the recipient tumour must be a true neoplasm; (iii) the metastatic neoplasm should show established growth in the recipient tumor, not the result of contiguous growth from an adjacent tumour or tumour emboli; and (iv) tumours that have metastasized to the lymph nodes where lymphoreticular malignant tumours  already exist, are excluded [3]. Our case fulfilled these criteria.

Although Malignant melanoma (MM) is one of the most common metastatic brain tumors, MM-to-meningioma metastasis is extremely rare [4,5]. There are only 2 cases reported in literature.

Meningiomas are the most common, primary intracranial tumors that host metastatic cancers [6]. The metastases usually arise from breast malignancies (women) and lung malignancies (men) [7,8]. There are several theories as to the predisposition for meningiomas to metastatic deposition. The rich vascular supply that may act as a vascular filter or the low flow rate within the cranial venous system, may increase the likelihood of tumour cell adherence and therefore metastasis [9]. Others have suggested that there is an absence of the host immune response within meningiomas thus increasing the chance of metastasis being located there compared to other primary brain tumours [4]. The exact cause for the propensity to metastatic deposition in benign tumours remains uncertain and is probably a combination of immunological and molecular factors within both the meningioma and the metastatic tumour.

In conclusion, this case does highlight the need to consider the presence of melanoma in intracranial and other tumours which may appear indolent or benign but whose biological behaviour appears to rapidly alter. This can lead to marked deterioration in the patients condition with rapid onset of neurological symptoms and marked oedema with the apparently “benign lesion” showing a rapid change in growth pattern.
